# Study of the Electrooxidation of a Zinc Concentrate

**DOI:** 10.3390/ma14112868

**Published:** 2021-05-27

**Authors:** Nora A. Tafoya-Medina, Cristina Chuck-Hernandez, Dora I. Medina

**Affiliations:** 1 Department of Process Engineering and Hydraulic, Universidad Autónoma Metropolitana-Iztapalapa, Ciudad de Mexico 09340, Mexico; 2 Tecnologico de Monterrey, School of Engineering and Sciences, Atizapan de Zaragoza, Estado de Mexico 52926, Mexico; 3 Department of Chemical Engineering, Instituto Politecnico Nacional-ESIQIE, Ciudad de Mexico 07738, Mexico; 4 Tecnologico de Monterrey, School of Engineering and Sciences, Monterrey 64849, Mexico; cristina.chuck@tec.mx

**Keywords:** electroleaching, zinc sulfides, sphalerite, pyrite

## Abstract

Zinc has wide industrial applications; consequently, its extraction procedures have been extensively studied. Hydrometallurgy is one of the most common methods employed for zinc recovery. However, the electrooxidation of sphalerite and the effect of the pyrite content in the concentrate have not been investigated; thus, in this work, zinc recovery from low-iron sphalerite mineral with a relatively high pyrite content (EBHSS), in a sulfate medium was further explored. The reaction mechanism of the anodic dissolution of the EBHSS mineral was established by microelectrolysis using mineral carbon paste electrodes; these results were used to determine adequate conditions for the macroelectrolysis of the sample. The macroelectrolysis indicated that EBHSS has a low electrodissolution rate; additionally, different analyses of the species produced in the macroelectrolysis showed that the ohmic drop registered in the collector had no influence in the passivation of the EBHSS surface. It was also determined that the dissolution of EBHSS was driven by the charge transfer of the sphalerite particles, which are not very efficient for electronic conductivity. Experiments using doped EBHSS led to an increase of the electrodissolution rate, which consequently increased the recovered zinc.

## 1. Introduction

Zinc is an important base metal required for various industrial applications [1]. It is mainly recovered from primary sulfide concentrates, sphalerite (ZnS) being the primary ore [2,3]. The sphalerite composition varies depending on the sulfide mineral source and is commonly associated with other sulfides such as chalcopyrite, galena and pyrite [4,5]. Methods for zinc recovery comprise mainly pyrometallurgical and hydrometallurgical processes [1,6,7]. Pyrometallurgical processes have traditionally been used for recovering metal from mineral concentrates, since these processes have metal recovery efficiencies of up to 99%. These methods involve several steps, including roasting, carbothermic reduction, reduction of sulfide ores, or metallothermic reduction, depending on the ore composition [8,9]. However, the production of sulfur dioxide and solid particles suspended in the air during combustion make these processes unfriendly to the environment [10,11].

The hydrometallurgical processes, on the other hand, are more environmentally friendly and rely on the use of aqueous solutions to decompose the sphalerite, forming an aqueous zinc sulfate electrolyte which is electrowon as metallic zinc at the cathode.

A significant consideration during the hydrometallurgical process is the reaction between sphalerite and ferric ion; consequently, the leaching reactions in ferric sulfate media [12,13] and ferric chloride media [14,15,16,17] have been widely studied. Using H_2_SO_4_ for zinc extraction is also a well-known method [18,19,20,21]. An important problem during the leaching step is the formation of elemental sulfur, which induces the passivation of metals in sulfide ores [22,23,24].

Therefore, it is necessary to investigate other alternatives that allow overcoming the limitations of hydrometallurgical processes in terms of passivation caused by the formation of sulfur species, during the initial stages. In this work an electrochemical strategy is proposed to study the electrooxidation of a sphalerite concentrate (zinc sulfide), using an applied electric potential as the oxidant. To determine the optimal pH and electrical potential, microelectrolysis assays were carried out; subsequently, the massive electrodissolution (macroelectrolysis) of the mineral was tested using a tridimensional electrochemical reactor for experimentation.

## 2. Materials and Methods

### 2.1. Sample Description

The two zinc sulfide concentrates (sphalerite) with an average particle size of 37 µm, from the Bismarck (Chihuahua, Mexico) and Rey de Plata (Guerrero, Mexico) mining units, were provided by Industrias Peñoles, S.A. de C.V. for the micro- and macroelectrolysis experiments. The preliminary mineralogical composition (Table 1) identified the sphalerite from the Bismarck unit as an iron and zinc sulfide, with relatively high iron content in solid solution (EAHSS) besides 3 wt.% pyrite (Industrias Peñoles, Torreon, Coahuila, México, pers.com). The sample from Rey de Plata was identified as a zinc sulfide with relatively low iron content in solid solution (EBHSS) besides 6 wt.% pyrite (Industrias Peñoles, Torreon, Coahuila, México, pers.com).

### 2.2. Microelectrolysis

To determine the electrochemical response of the sphalerite samples, a 100 mL Pyrex cell was used to perform the voltametric analyses. The lid of the glass cell had four orifices to place a three-electrode system (working, auxiliary and reference electrodes) and a N_2_ injection. The working electrode consisted of a plastic tube (length, 7 cm; diameter, 0.2 cm) with a piston, into which was introduced a mineral carbon paste prepared using a homogeneous mixture of 0.7 g of graphite and 0.3 g of either low-iron or high-iron sphalerite, agglomerated with oil silicone (electrode CPE-ZnS). The active area of the working electrode (3.14 × 10^−2^ cm^2^) was polished using a Carbimet Piper disc (Buhler 600, Grupo Mess, Monterrey, Mexico). The electrical contact was a silver-welded copper-platinum connection.

After each voltametric measurement, the piston re-established the effective area of the working electrode. Subsequently, its active area was levelled again, and the dissolved oxygen was removed from the working solution using a N_2_ flow. The auxiliary electrode was a graphite bar of 9 cm length, 0.6 cm diameter, and purity of 99.9995% (Alfa Aeser, Johnson Matthey, United States). The reference electrode was a saturated mercury sulfate electrode (SSE; Tacussel, Lyon, France), connected to the glass cell by a Luggin capillary.

An AUTOLAB potentiostat was used for experimental control and data acquisition of the high-iron (EAHSS) and low-iron (EBHSS) sphalerites. To determine the oxidation stages of the low-iron sphalerite (EBHSS), voltamperometric and chronoamperometric analyses were performed using the AUTOLAB software GPES (General Purpose Electrochemical System, version 4.9, The Netherlands), delivered with the AUTOLAB potentiostat. The voltammograms were obtained in solutions of Na_2_SO_4_ (0.1 M, pH = 7), and H_2_SO_4_ (0.1 M, pH = 2; 1.7 M), with no electrolyte agitation, over a range of −2 ≤ E ≤1 and at a scan speed of 100 mV each for 1 s, using CPE-ZnS samples (70:30 wt.%). The solutions were prepared using deionized water with a specific resistance of 18.2 mΩ^−1^ cm^−1^ and analytic-grade reagents. Scanning electron microscope analyses (SEM; Jeol JSM 6300, MA, United States) were conducted to study the mineral passivation.

### 2.3. Macroelectrolysis

Based on the oxidation stages detected during microelectrolysis of the EBHSS sphalerite, a parallel flow tri-dimensional nylamide electrochemical reactor (1 L) was used for the macroelectrolysis experiments. The working electrode was a graphite cloth containing 10 g of homogeneously distributed EBHSS sphalerite. The auxiliary electrode was prepared using five stainless steel meshes (9 cm diameter, mesh 100). The reference electrode was a saturated sulfate electrode (SSE; Tacussel) connected to the electrochemical reactor by a Luggin capillary.

The chronopotentiometric data were collected using an EG&G potentiostat (Princeton Applied Research, Houten, The Netherlands). For measurement of the voltametric and chronoamperometric data, an AUTOLAB potentiostat was used. For the chronoamperometric study, the applied potential (E_λ+_) range was 0.4 ≤ E_λ+_ ≤ 1.0 V, with pulse of 180 s. The experimental solutions of Na_2_SO_4_ (0.1 M, pH = 7) and H_2_SO_4_ (0.1 M, pH = 2; 1.7 M) were prepared using analytical-grade reagents.

Atomic absorption analysis were carried out to determine the chemical composition of the EBHSS sphalerite, and to quantify the electro-dissolved metallic ions, using a Varian atomic absorption spectrophotometer (SpectrAA-20, Manasquan, NJ, United States). The surface morphology of EBHSS after electroleaching was analyzed using a scanning electron microscope (SEM; Jeol JSM 6300).

## 3. Results and Discussion

### 3.1. Electrochemical Response of EAHSS and EBHSS

**Figure 1:**
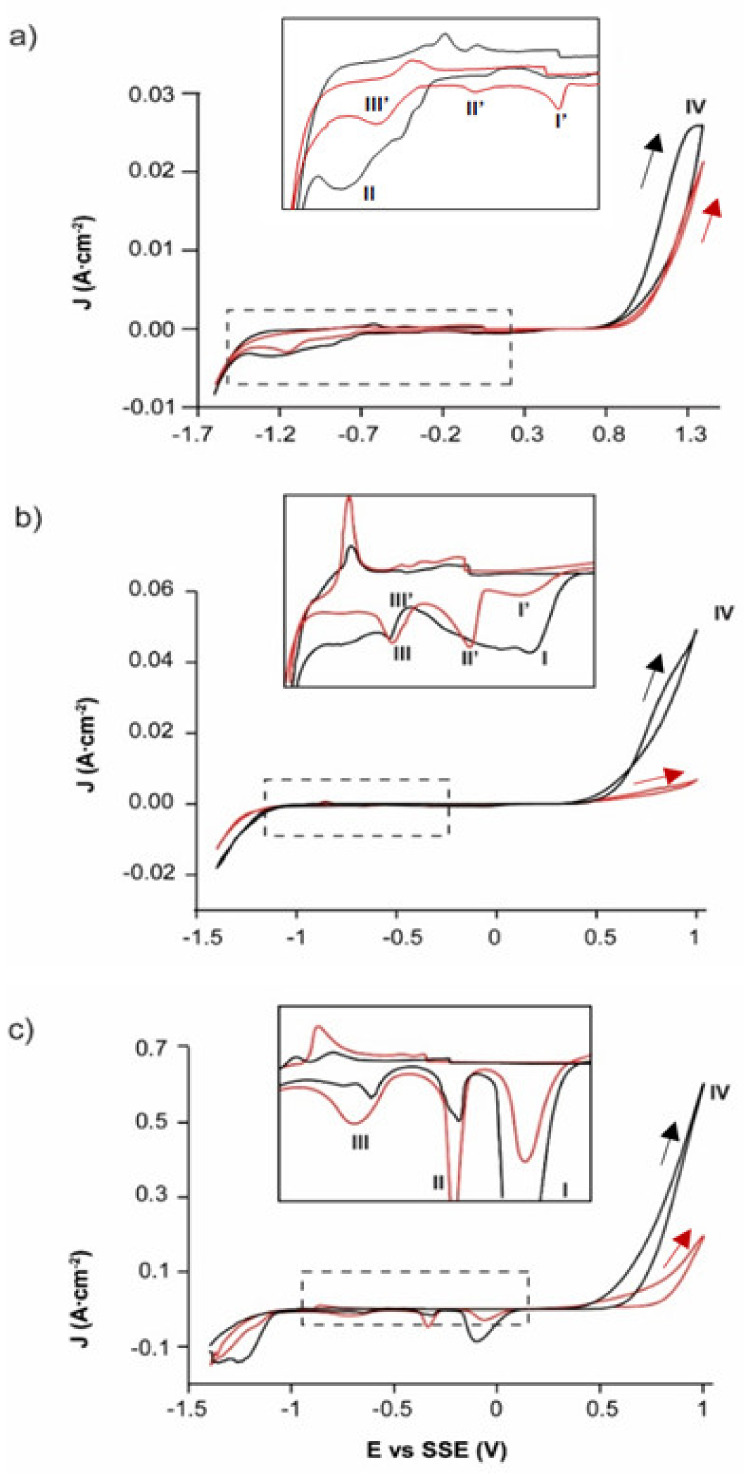
Typical CPE-ZnS voltamperograms (70:30 wt.%) obtained at a scan speed of 100 mVs^−1^ and positive direction. (**a**) 0.1 M Na_2_SO_4_, pH = 7; (**b**) 0.1 M H_2_SO_4_, pH = 2; (**c**) 1.7 M H_2_SO_4_. EAHSS, red line; EBHSS, black line. Full line squares show the detailed redox processes of the samples indicated in dashed squares.

The cyclic voltammetry analyses for comparing the electrochemical response to the oxidation of the EAHSS and EBHSS concentrates indicated that EAHSS and EBHSS presents an oxidation process, associated with the oxidation of sphalerite (Figure 1). However, EBHSS presented more significant redox processes than EAHSS under the same pH and concentrations. Conversely, the EBHSS electrodissolution speed was higher than that of EAHSS at any given pH and concentration, generating a significant oxidation current density. At pH = 7 (Figure 1a), the reduction process I was observed in EAHSS, which could not be identified for EBHSS. At pH = 2 (Figure 1b), the process I of EBHSS combines the reduction processes I’ and II’ of EAHSS. These reduction processes can be complex so they are not studied intensively in this work.

For an electrolytic medium of H_2_SO_4_ (1.7 M), the reductions of EBHSS and EAHSS were different only in the value of their respective cathodic current peaks (Figure 1c). It was noted that, as the electrolyte pH decreased, the associated oxidation current density of the oxidation process IV increased, generating an elevated oxidation current density, which was associated with a higher concentration of the ion H^+^. Narasagoudar et al. [25] showed that during the chemical dissolution of the sphalerite the associated oxidation current density (J) increased as the electrolyte pH decreased due to the presence of the ion H+. A substantial increase in the associated oxidation current density caused by the formation of H_2_S during the anodic dissolution was also reported (reaction (1)):

ZnS + 2H^+^_(aq)_ 
➔ Zn^2+^ + H_2_S_(aq)_ (1)

### 3.2. Effect of Pyrite Content in EBHSS and EAHSS

Analyzing the relative iron-content influence in the redox behavior of the sphalerite samples (EBHSS and EAHSS), it was found that there is a more important oxidation process in EBHSS than in EAHSS. The electrodissolution of two zinc concentrates with different content of iron in solution (0.8% and 12.3%) was studied by Ahlberg et al. [26]. The results showed that the concentrate with a low amount of iron in solution did not present an electrochemical response which suggests that the dissolution rate depends on the amount of iron present in the solid solution with the zinc concentrates.

However, the iron content in both zinc concentrates is present as solid solution and as pyrite, which can act as a galvanic couple of sphalerite. Mehta and Murr [27] observed that the leaching speed of the sphalerite increased according to the increase of pyrite in contact with sphalerite. Therefore, they suggested that when two sulfide minerals are present in an acid aqueous solution, the less noble sulfide will easily dissolve, whereas the nobler sulfide will be galvanically protected. Cruz et al. [28] also showed that sphalerite can act as a galvanic protection to pyrite. For example, during leaching, pyrite will become more reactive as sphalerite is dissolved. However, in this study, the effect of pyrite on sphalerite was probably masked by the overpotential applied (1 V) to the system.

**Figure 2:**
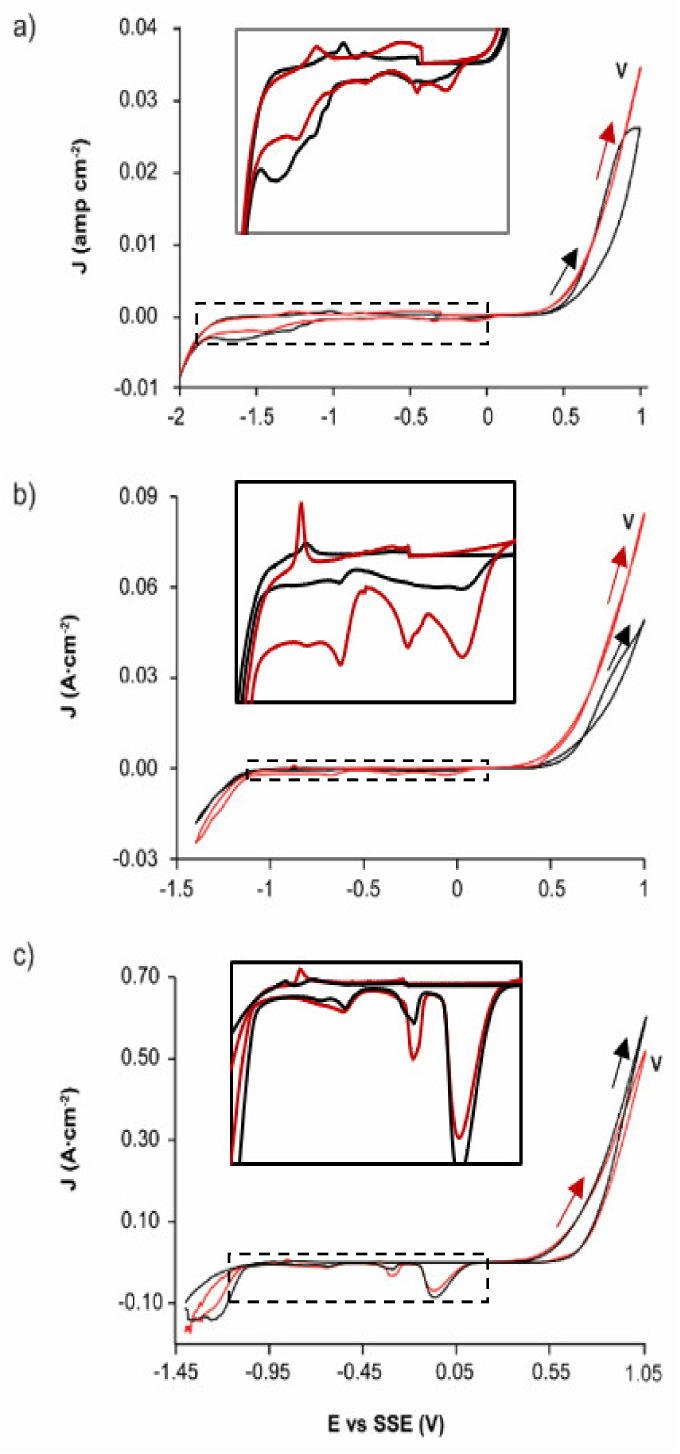
Typical CPE-ZnS voltamperograms (70:30 wt.%) obtained at a scan speed of 100 mVs^−1^ and positive direction. (**a**) 0.1 M Na_2_SO_4_, pH = 7; (**b**) 0.1 M H_2_SO_4_, pH = 2; (**c**) 1.7 M H_2_SO_4_. Red line indicates EAHSS with 10 wt.% pyrite; black lines, EBHSS. Full line squares show the detailed redox processes of the samples indicated in dashed squares.

On the other hand, the oxidation process of EBHSS was relatively more significant than the oxidation process of EAHSS because the pyrite oxidation also occurred in the same potential range (Figure 1). To test this, voltametric analyses were performed, at the same pH and concentration conditions, using the working electrode (CPE-ZnS) prepared with EAHSS and an additional 10 wt.% of pyrite. The pyrite addition increased the associated oxidation current density (J) at any pH and concentration (peak V). These results suggested that the presence of pyrite in the EBHSS samples also contributed to its electrooxidation when elevated potentials such as 1 V were applied at pH = 7 (Figure 2a); pH = 2 (Figure 2b) and 1.7 M H_2_SO_4_ (Figure 2c).

It is important to highlight that the peaks I’, II’, III’ of Figure 1, associated with the mineral EAHSS become more evident in Figure 2 with the addition of 10 wt.% pyrite, which indicates that these cathodic processes correspond to the reduction of the species formed during the anodic oxidation of the pyrite present in the zinc concentrate, specifically iron hydroxides [29], and some iron polysulfides [30].

According to the results, the EBHSS mineral presents a greater current gain under the applied potential range, due to the oxidation of pyrite, a mineral found in appreciable quantities in this zinc concentrate. For its part, the electrochemical response of the mineral EAHSS is mainly due to the oxidation of sphalerite since pyrite is present in smaller quantities.

### 3.3. Oxidative Dissolution of EBHSS

**Figure 3:**
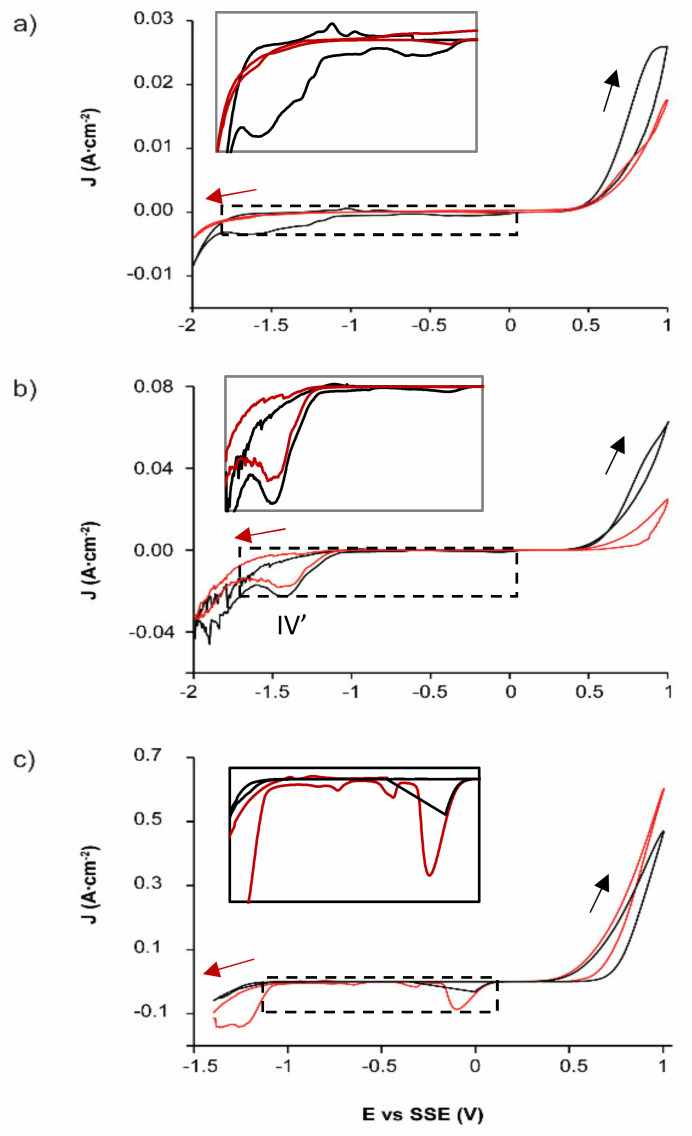
Typical CPE-ZnS voltamperograms (70:30 wt.%) obtained at a scan speed of 100 mVs^−1^, without electrolyte agitation for EBHSS. (**a**) 0.1 M Na_2_SO_4_, pH = 7; (**b**) 0.1 M H_2_SO_4_, pH = 2; (**c**) 1.7 M H_2_SO_4_. The red line indicates negative direction; black line, positive direction. Full line squares show the detailed redox processes of the samples indicated in dashed squares.

When the voltametric analyses are performed in the positive direction (Figure 3), EBHSS showed reduction processes, in contrast to the analyses in the negative direction, where such processes were not observed. A notable exception occurs at pH = 2 (Figure 3b), when the reduction process IV’ occurs in both positive and negative directions. The largest responses were obtained when the voltammetry starts in the positive direction at any pH (Figure 3a–c), corresponding to the generation of some species (i.e., iron hydroxides) that can partially passivate the mineral surface when they are reduced (Table 2). In addition, pyrite oxidation results in cathodic processes in EBHSS (Figure 1 and Figure 2); therefore, it is important to differentiate the EBHSS oxidation from the pyrite oxidation.

**Figure 4:**
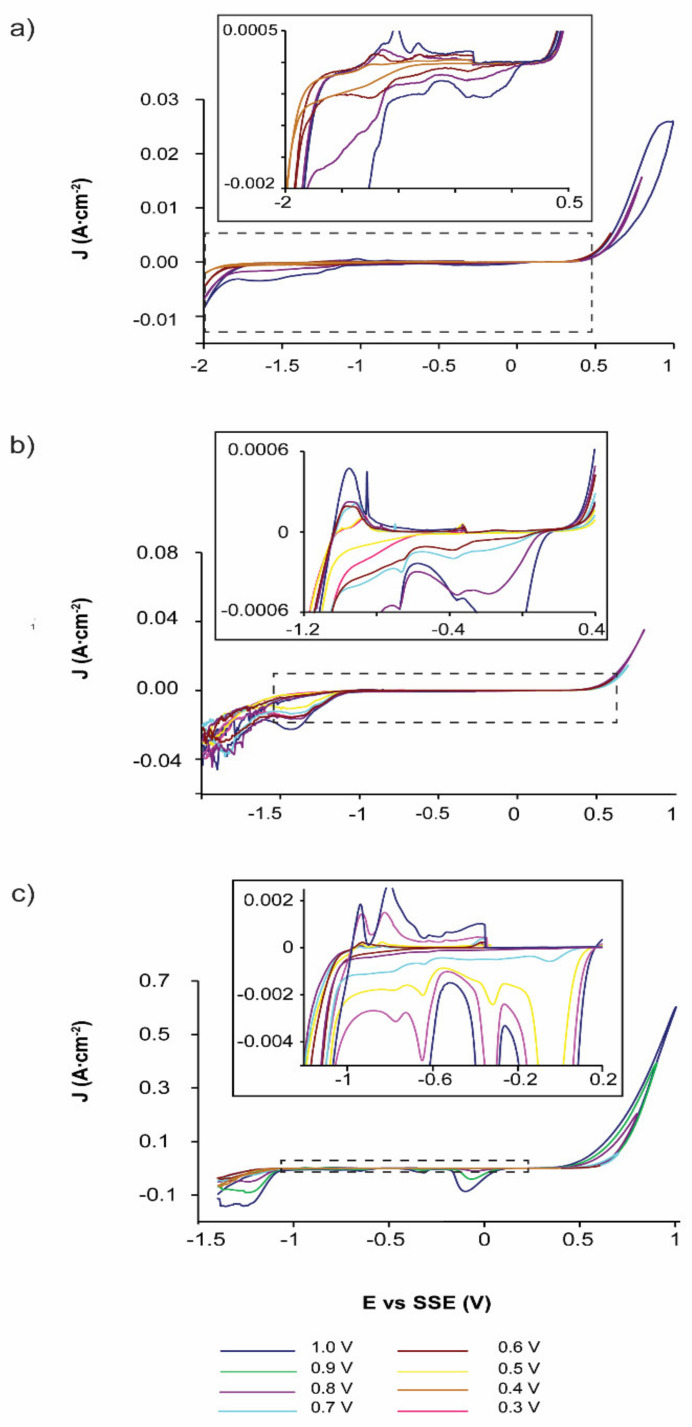
Typical CPE-ZnS voltamperograms (70:30 wt.%) obtained at a scan speed of 100 mVs^−1^. The scan was initiated in the positive direction. (**a**) 04.1 M Na_2_SO_4_, pH = 7; (**b**) 0.1 M H_2_SO_4_, pH = 2; (**c**) 1.7 M H_2_SO_4_. The colors indicated different values of inversion anodic potential. Full line squares showed the detailed redox processes indicated in dashed squares.

Regarding the effect of inversion anodic potential (E_λ+_), Figure 4 shows that the anodic and cathodic currents are potential dependent, meaning that when the potential (E_λ+_) increases, the current associated to the oxidation-reduction processes also increases.

At pH = 7 (Figure 4a), the reduction processes become imperceptible when the inversion potential is E_λ+_ = 0.6 V. At pH = 2 (Figure 4b), pyrite oxidation occurs when the inversion potential is E_λ+_ > 0.5 V. Using a 1.7 M H_2_SO_4_ electrolyte (Figure 4c), the current reduction decreases at an inversion anodic potential of 0.6 V. Therefore, pyrite oxidation notably occurs at E_λ+_ > 0.5 V; however, at lower E_λ+_ values, the sphalerite oxidation is predominant.

Table 2 shows the charge density associated with the oxidation peaks, Qa (when the sweep starts in a positive direction) and Qa’ (when the sweep starts in a negative direction). The highest charge densities are obtained when starting the sweep in the positive direction, for any pH value. This could indicate that when starting the sweep in the negative direction, species are generated that partially passivate the mineral surface.

**Figure 5:**
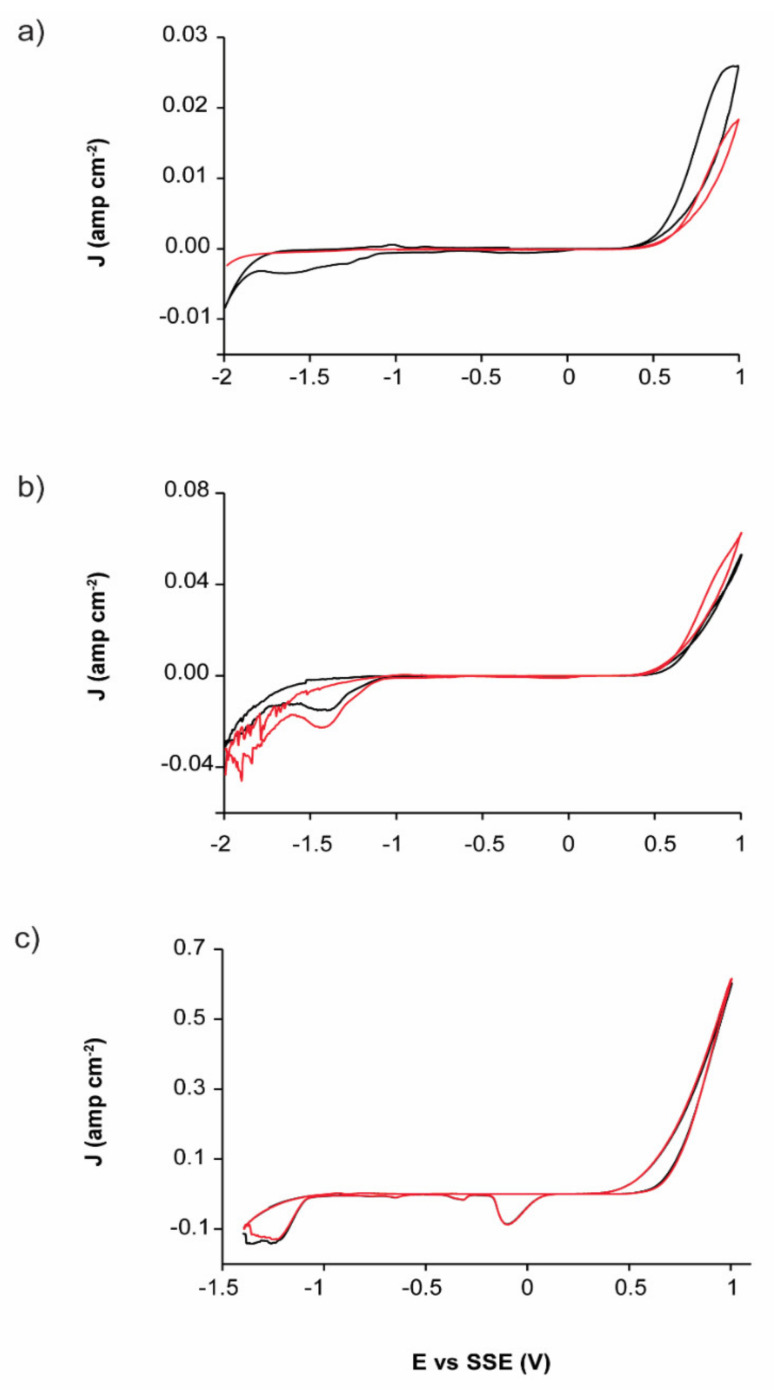
Typical CPE-ZnS voltammograms (70:30 wt.%) obtained at a scan speed of 100 mVs^−1^. The scan was initiated in the positive direction. (**a**) 0.1 M Na_2_SO_4_, pH = 7; (**b**) 0.1 M H_2_SO_4_, pH = 2; (**c**) 1.7 M H_2_SO_4_. The red lines indicate voltammetry without agitation; black lines, voltammetry with agitation.

At pH = 7 (Figure 5a), the products of the EBHSS oxidation are soluble. When the electrolyte is agitated, the chemical species attached to the working electrode surface are removed; consequently, they cannot be reduced during the reverse scan. At pH = 2 (Figure 5b), the reduction of EBHSS decreases substantially with agitation. This can be explained by the fact that, although the oxidation products (i.e., sulfates, thiosulfates) are soluble [31], the agitation does not remove them completely from the electrode-solution interface when they are present at elevated concentrations. When the 1.7 M H_2_SO_4_ electrolyte is used (Figure 5c), the electrochemical behavior of EBHSS is practically the same with or without agitation because its oxidation products are species that can be attached to the electrode surface. These results indicate that, as the pH decreases, the associated current density (J) increases; therefore, an increase in the proton concentration promotes the anodic dissolution of EBHSS. According to Nava [32], such behavior can be attributed to the simultaneous electro-dissolution and the chemical dissolution of sphalerite. Furthermore, the oxidation of the produced H_2_S can contribute to the electro-dissolution of sphalerite. 

**Figure 6:**
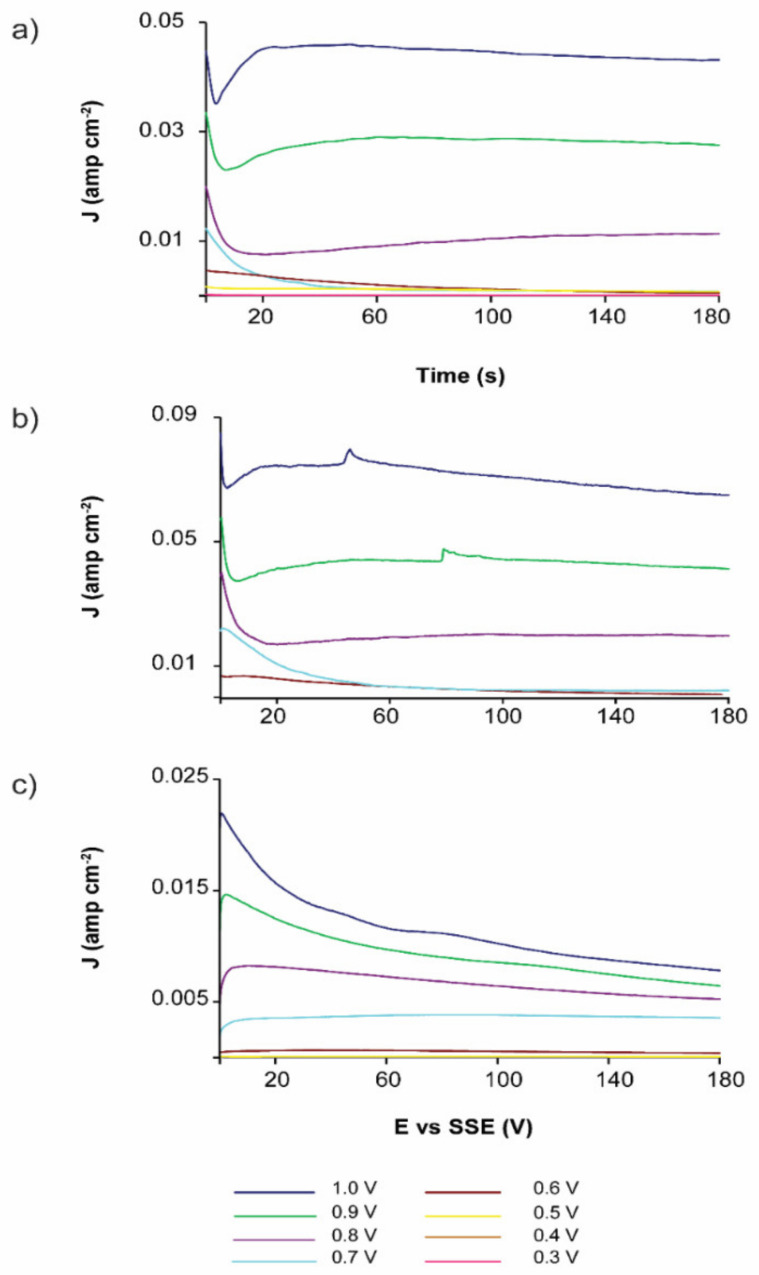
Typical current transient of CPE-ZnS voltamperograms (70:30 wt.%) obtained without electrolyte agitation. (**a**) 0.1 M Na_2_SO_4_, pH = 7; (**b**) 0.1 M H_2_SO_4_, pH = 2; (**c**) 1.7 M H_2_SO_4_. The colors indicate different potentials applied.

The oxidation of EBHSS for different electrooxidation potentials is shown in Figure 6. At pH = 7 and pH = 2, the curve related to the oxidation potential, E_λ+_ = 0.70 V, showed a decrease related to the oxidation of solid species (S^0^) at the working electrode-electrolyte interface. At potentials above 0.7 V, the passivation process disappears due to the transformation of elemental sulfur to more elevated oxidation states.

**Figure 7:**
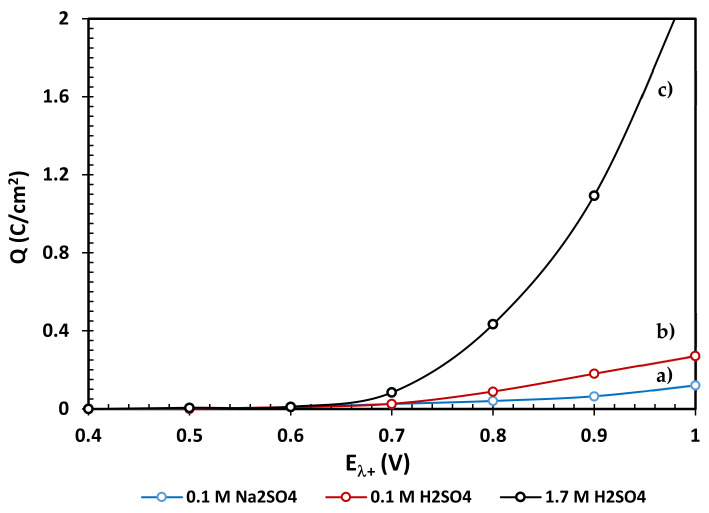
Variation of the voltametric oxidation charge densities of the zinc concentrate EBHSS, as a function of the anodic applied potential, at different pH values, (**a**) 0.1 M Na_2_SO_4_, pH = 7, (**b**) 0.1 M H_2_SO_4_, pH = 2, (**c**) 1.7M H_2_SO_4_. Analyses performed without agitation.

**Figure 8:**
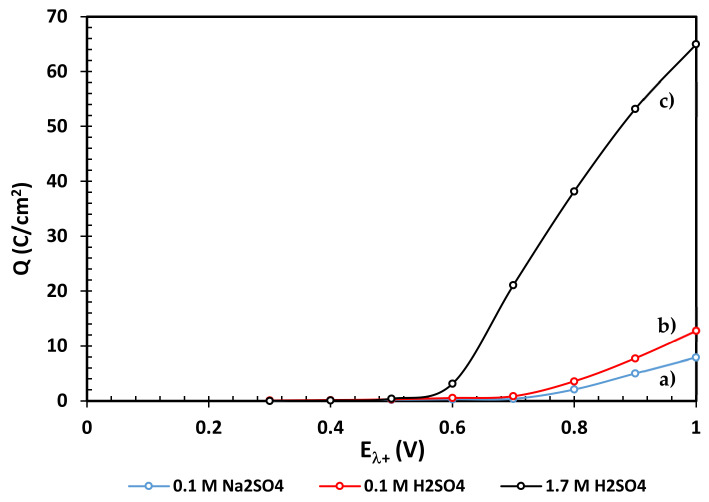
Chronoamperometric charge densities for the zinc concentrate EBHSS using several electrolytic media at different pH values, (**a**) 0.1 M Na_2_SO_4_, pH = 7, (**b**) 0.1 M H_2_SO_4_, pH = 2, (**c**) 1.7 M H_2_SO_4_. Analyses performed without agitation.

The evaluated charge densities from the voltammetries (Figure 4) and chronoamperometries (Figure 6) are shown in Figure 7 and Figure 8, respectively, where it can be clearly identified that the oxidation stages of the EBHSS mineral are dependent on the applied potential. The charge densities associated to the oxidation process of EBHSS at different pH and concentrations (Figure 7) did not show significant changes at inversion potentials of E_λ+_ < 0.7 V. A significant increase of transformed species (i.e., S^2−^) during the anodic dissolution was also observed at E_λ+_ > 0.7 V.

According Figure 8, the anodic dissolution of the concentrate increases significantly after 0.7 V for all electrolytic media. For pH = 7 and pH = 2, a drop in charge density (Q) is observed when the applied potential is 0.7 V, which indicates a passivation process of the mineral surface, which inhibits its electrodissolution. At 1.7 M this passivation behavior does not occur, however the charge density increases substantially at potentials greater than 0.6 V, showing an exponential increase in its electrodissolution rate.

On the other hand, the voltametric and chronoamperometric analyses showed that the oxidation stages of EBHSS are dependent on the potential (E_λ+_). The oxidation reactions related to such processes are:

Oxidation reactions of EBHSS at pH = 7

Anodic potential E_λ+_ ≤ 0.7 V

ZnS → Zn^2+^ + S^0^ + 2e^−^ (2)

E* = −0.526 V vs. SSE

At E_λ+_ ≤ 0.7 V, a passivation process associated to the development of an elemental sulfur layer occurs on the EBHSS surface. This process can be considered a thermodynamically independent stage at any positive pH value.

• Anodic potential E_λ+_ ≥ 0.7 V

When an elevated overpotential (1 V) is applied to the EBHSS oxidation process, it is significantly modified because the sulfides are oxidized to higher oxidation states such as thiosulfates or sulfates. The elevated overpotential allows the oxidation of S_2_O_3_^−2^ to SO_4_^−2^ since E* in Equation (3) is less positive than the value required in Equation (2).

2ZnS + 3H_2_O ➔ 2Zn^2+^ + S_2_O_3_^2−^ + 6H^+^ + 8*e* (3)

E* = −0.547 V vs. SSE

S_2_O_3_^2−^ + 5H_2_O − 8e^−^➔2SO_4_^2−^ + 10H^+^ (4)

E* = −0.9215 V vs. SSE

Oxidation reactions of EBHSS at pH = 2

The oxidation stages of EBHSS are practically the same at pH = 2 as at pH = 7; however, the conditional potential will change when elevated overpotentials are applied and in systems where pH has a remarkable influence.

• Anodic potential E_λ+_ ≤ 0.7 V

ZnS ➔ Zn^2+^ + S^0^ + 2^e−^ (5)

E* = −0.526 V vs. SSE

• Anodic potential E_λ+_ ≥ 0.7 V

2ZnS + 3H_2_O ➔ 2Zn^2+^ + S_2_O_3_^2−^ + 6H^+^ + 8e^−^ (6)

E* = −0.3258 V vs. SSE

S_2_O_3_^2−^ + 5H_2_O − 8e^−^ ➔ 2SO_4_^2−^ + 10H^+^ (7)

E* = −0.556 V vs. SSE

Oxidation reactions of EBHSS at 1.7 M H_2_SO_4_

Using the 1.7 M H_2_SO_4_ electrolyte, the EBHSS redox processes dramatically change because the chemical species produced during the oxidation of EBHSS can be attached to the electrode surface. In addition, the oxidation stages of EBHSS cannot be clearly distinguished due to the passivation process occurring at E_λ+_ = 0.7 and different pH.

Oxidation reactions of pyrite at pH = 2 and pH = 7

Different experiments have suggested that sphalerite and pyrite oxidation can simultaneously occur. This study indicates that pyrite oxidation follows a reaction pathway where ferric ions or sulfates (at acid pH conditions and elevated oxidation potentials) are produced [33].

FeS_2_ + 8H_2_O ⇿ Fe^2+^ + 2SO_4_^2−^ + 16H^+^ + 14e^−^ (8)

The formation of intermediate species such as iron polysulfides has also been reported (reaction (9)), which turns the colorless electrolyte yellow [30]. This reaction tends to occur in greater proportion in alkaline media because such chemical species are more stable reaction products at elevated pH than under acidic conditions.

FeS_2_ ⇿ Fe_1−x_ S_2−y_ + xFe^3+^ yS + 3xe^−^ (9)

In addition, when pyrite oxidizes (reaction (8)), the ferric ion can produce iron hydroxides (reaction (10)). On the other hand, when in solution iron, Fe^2+^, and zinc, Zn^2+^ are released at the same time, iron may follow the reaction pathway shown in reaction (7) to produce iron hydroxides.

Fe^2+^ + 3H_2_O ⇿ Fe(OH)_3_ + 3H^+^ + 1e^−^ (10)

### 3.4. Macroelectrolysis I

**Figure 9:**
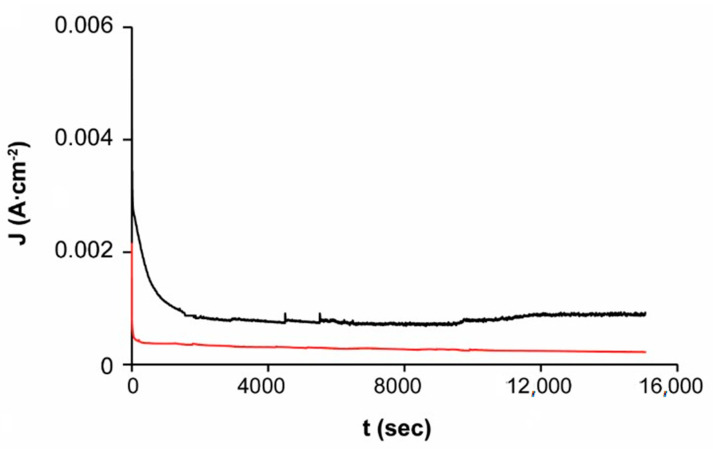
Electrolysis of EBHSS at constant potential E_λ+_ = 1 V vs. SSE of direct current. The red line indicates pH = 7; black line pH = 2.

The chronoamperometric study of the sphalerite EBHSS at 25 °C, pH = 7 and pH = 2 is shown in Figure 9. At pH = 2, the electro-dissolution speed is double the speed obtained at pH = 7, which is consistent with the results of the microelectrolysis analyses. These results suggest that acid conditions are the most advantageous for the electro-dissolution of EBHSS.

During the macroelectrolysis at pH = 7, intermediate iron species such as polysulfides could have been formed from the pyrite oxidation because the colorless electrolyte turned yellow [30]. Conversely, using an electrolyte of elevated acidity (pH = 2), the yellow color was not observed; therefore, the intermediate species were not formed.

**Figure 10:**
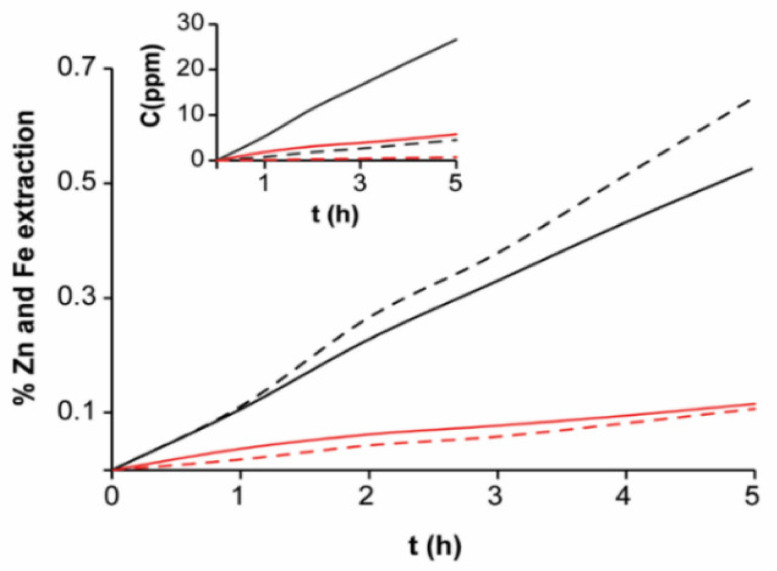
Percentage of extracted zinc and iron related to the initial sphalerite sample concentration (Zn, 5 g; Fe, 0.7 g) at 25 °C. Red lines show the extraction at pH = 7; black lines, at pH = 2. Full lines represent Zn; dashed lines, Fe. The inserted plot indicates the concentration changes of zinc ions and ferrous ions.

Changes in the electroleached zinc and iron at pH = 2 and 7 in function of electroleaching time and total initial concentration are shown in Figure 10. The current efficiency for zinc in EBHSS was calculated from reaction (2), which takes into consideration that the zinc oxidation can transfer up to eight electrons to produce thiosulfates and sulfates. At pH = 2, the current efficiency of the electrolysis was 68%, whereas at pH = 7, the current efficiency was 61%, indicating that most of the current flowing through the electrodes originated from sphalerite oxidation. The chemical dissolution of EBHSS, without applying an anodic potential E_λ+_, occurs at pH = 2, which indicates that the amount of dissolved zinc via chemical dissolution is negligible compared to the amount of dissolved zinc obtained from EBHSS electroleaching.

**Figure 11:**
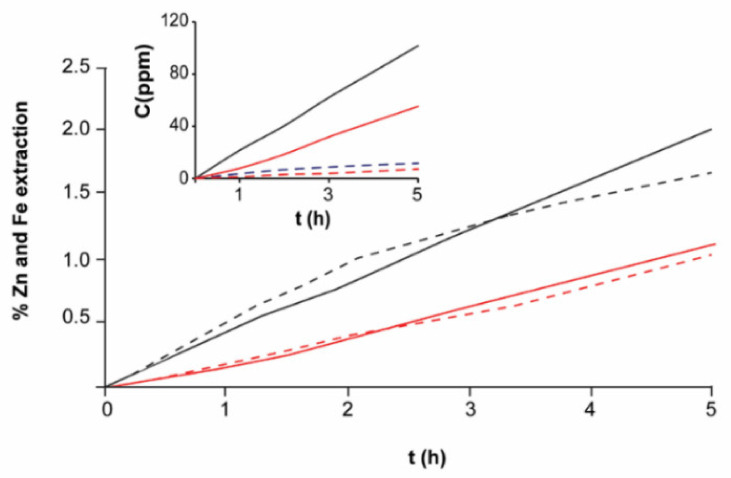
Percentage of extracted zinc and iron related to the initial sphalerite sample concentration (Zn, 5 g; Fe, 0.7 g) at 40 °C. Red lines show the extraction at pH = 7; black lines, at pH = 2. Full lines represent Zn; dashed lines, Fe. The inserted plot indicates the concentration changes of zinc ions and ferrous ions.

At 40 °C (Figure 11), the electroleaching speed of EBHSS increases significantly compared to the electroleaching process at 25 °C (Figure 10), in a proportion of 1:4 for pH = 7 and 1.5:1 for pH = 2. The previous data suggest that the temperature influences the semiconducting properties of the EBHSS. Rius de Riepen and Castro Acuña [34] attributed this behavior to the excitation produced to the valence electrons using either a thermic or luminous energy; the excitation causes the electrical conductivity to be directly proportional to temperature; therefore, the conductivity will increase 5% for every degree Celsius.

The percentage of extracted zinc from the total content of EBHSS is shown in Figure 11. It is shown that zinc recovery is approximately four times greater at 40 °C than at room temperature. At pH = 2, the efficiency of the oxidation current of the zinc was 63%; at pH = 7, it was 60%. Therefore, the efficiency of zinc oxidation is elevated, although the zinc recovery rate is low, indicating that the zinc electrodissolution is a slow process compared to the commercial process of Sherrit Gordon Mines Limited [35,36].

**Figure 12:**
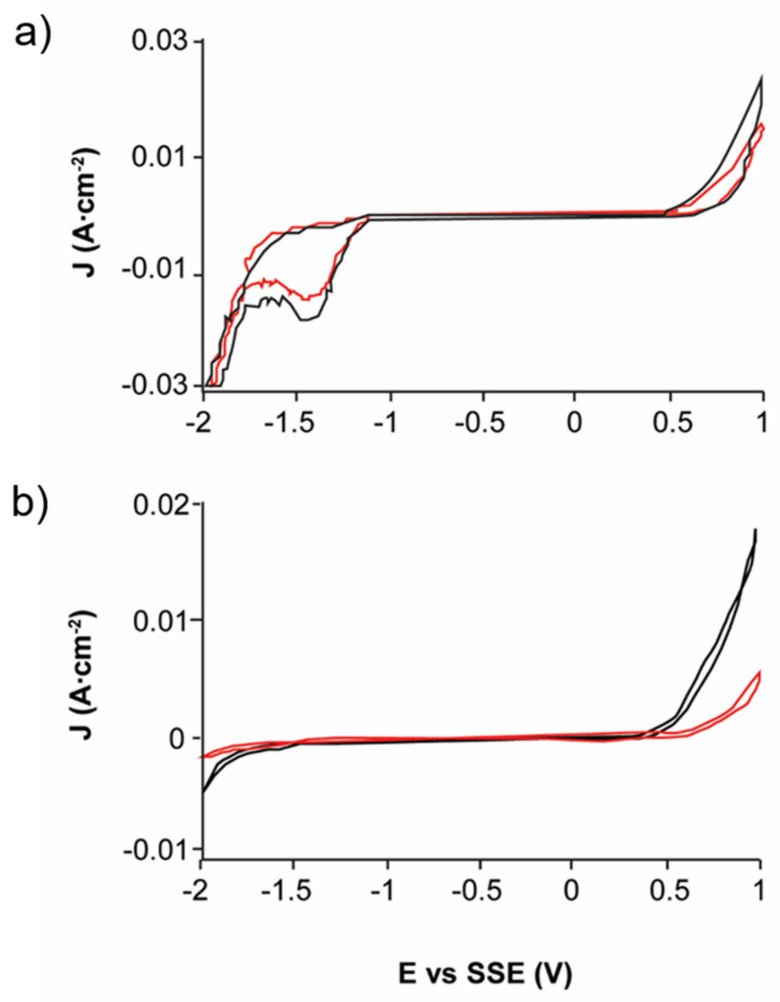
Typical CPE-ZnS voltamperograms (70:30 wt.%) obtained at a scan speed of 100 mVs^−1^, without electrolyte agitation and negative direction. (**a**) 0.1 M H_2_SO_4_, pH = 2; (**b**) 0.1 M Na_2_SO_4_, pH = 7. The black line shows the signal before electrolysis; red line, after electrolysis.

The scanning in the negative direction of both untreated EBHSS and EBHSS after electrolysis indicates that, at pH = 7 and pH = 2 (Figure 12), the electrochemical behavior of the EBHSS did not show a significant difference and the presence of elemental sulfur on the mineral surface was not detected; therefore, the redox reactions occur until the formation of soluble sulfur species.

**Figure 13:**
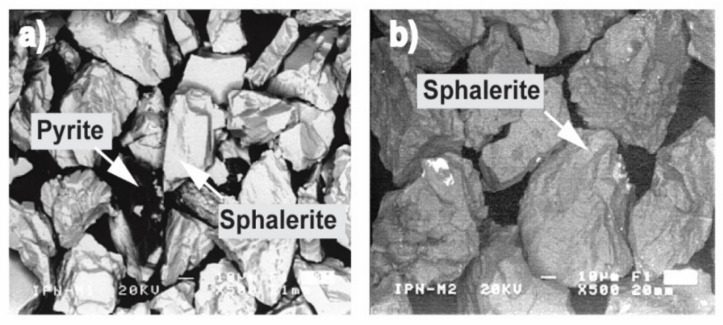
Surface morphology of sphalerite particles (**a**) before electrolysis; (**b**) after electrolysis.

**Figure 14:**
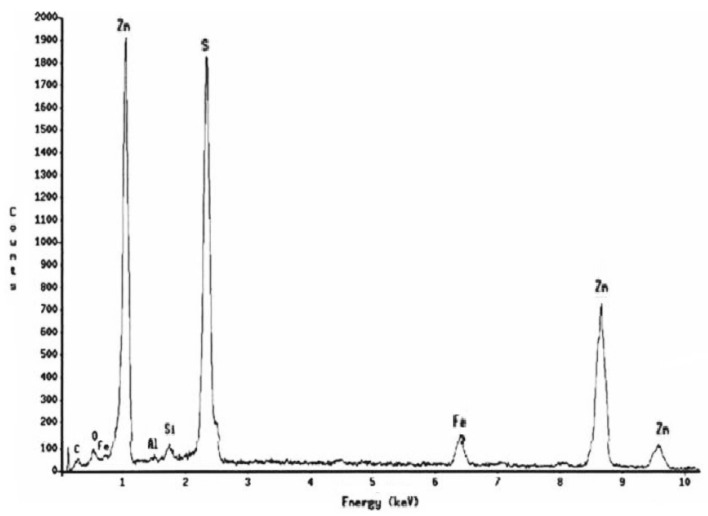
Microanalysis of sphalerite particles shown in Figure 13, before electrolysis.

**Figure 15:**
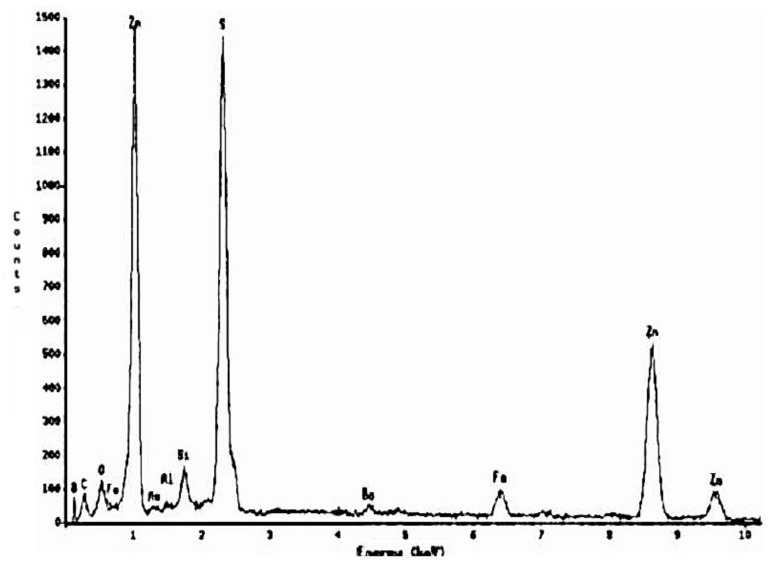
Microanalysis of sphalerite particles shown in Figure 13, after electrolysis.

The Figure 13 shows that, at pH = 2, the surface morphology of the EBHSS does not change before and after electroleaching. In addition, the presence of a passivating specie (S^0^) attached to the EBHSS surface was not observed. The intensity changes of Zn, Fe, and S peaks related to the electrolytic recovering changes before and after electroleaching (Figure 14 and Figure 15), indicating that a portion of these elements has been recovered during the electroleaching.

**Figure 16:**
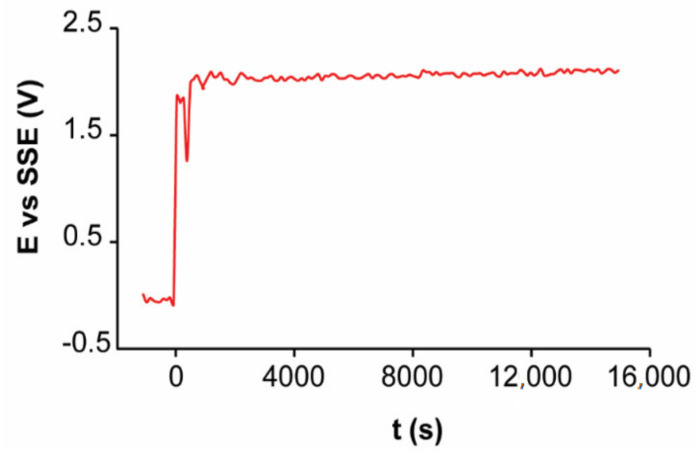
Electrolysis of EBHSS using a current intensity of 100 mA at pH = 2 and T = 25 °C.

On the other hand, the chronoamperometric analyses (Figure 16) show an initial transition time in which the current density is used for charging the double layer. Subsequently, a slight potential variation in function of time is observed, reaching a constant potential difference over time.

The comparison of the chronopotentiometric and chronoamperometric analyses of EBHSS (Table 3) indicates that, when a current of 100 mA is applied, the working electrode produces on average a potential of 2 V vs. SSE. Conversely, a potential of 1 V vs. SSE produces a current of 64 mA, showing a significant ohmic drop in the working electrode. According to the results of the microelectrolysis, the slow kinetic electrodissolution of EBHSS may be associated with its low electrical conductivity [37] and the type of electrical contact that prevails in the working electrode, but specifically with the sphalerite particles (contact type n-n).

### 3.5. Macroelectrolysis II

**Figure 17:**
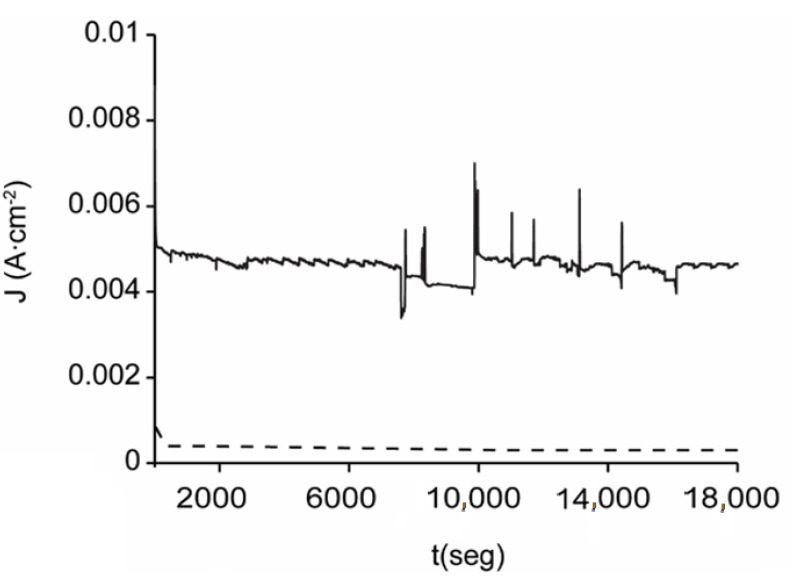
Electrolysis at constant potential of doped EBHSS, E_λ+_ = 1 V vs. SSE of direct current at pH = 2 and 25 °C. Full line indicates doped EBHSS; dashed line, pure EBHS.

Based on the results of the macroelectrolysis I, the EBHSS sample was doped (5% graphite). In comparison to the macroelectrolysis I results, the current of the macroelectrolysis II increased by a ratio of 1:10 and 1:5 at pH = 7 and pH = 2, respectively. However, as the current increased, the fisiadsorption of the hydrogen bubbles on the auxiliary electrode also increased, causing electrical fluctuations (Figure 17).

**Figure 18:**
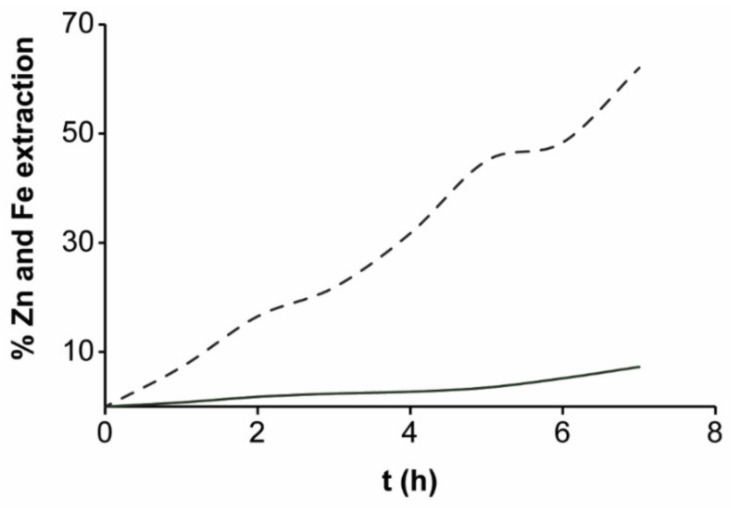
Percentage of extracted zinc and iron at 25 °C as related to the initial sphalerite sample (Zn, 5 g; Fe, 0.7g) doped with graphite. Full lines represent Zn; dashed lines, Fe.

Regarding the conversion of zinc and iron with respect to time, the iron concentration obtained in the macroelectrolysis II was higher than the zinc concentration, which suggests that when EBHSS is doped, the iron and zinc are simultaneously oxidized (Figure 18). Additionally, the doping of EBHSS enhanced the zinc and iron extraction up to 7% and 62%, respectively. For a 7-h macroelectrolysis, most of the iron content in the EBHSS was dissolved, with a current efficiency of 66%, but the reaction not only favors the zinc dissolution at a potential of 1 V vs. SSE. According to the microelectrolysis analyses, the application of potentials E_λ+_ < 1 V vs. SSE can minimize the pyrite electrodissolution but cannot avoid the dissolution of the iron arranged in solid solution with sphalerite.

## 4. Conclusions

In this work, the electrochemical response of two zinc sulphide concentrates was initially studied, one with a high iron content in solid solution (EAHSS) and the other with a low iron content in solid solution (EBHSS), both concentrates with an amount of iron as pyrite. The oxidation process is more important for EBHSS, because in the range of potential of study, in addition to the oxidation of the sphalerite, the oxidation of the pyrite also occurs, a mineral that is present in appreciable quantities in this zinc concentrate. For this reason, this mineral was chosen to continue with the study.

The electrochemical study allowed to establish the potential ranges where this zinc concentrate is oxidized. The microelectrolysis of EBHSS at pH = 2 and pH = 7 showed that oxidation occurs in two stages. The first stage was identified at E_λ+_ < 0.7 V vs. SSE, where sulfide is oxidized to elemental sulfur. The second stage of oxidation occurs at E_λ+_ > 0.7 V vs. SSE where the elemental sulfur is converted to sulfates and thiosulfates, although iron can be electroleached at any oxidation stage when it is presented in dissolution or at E_λ+_ > 0.6 V in the form of pyrite. However, when the reaction occurs at 1.7 M H_2_SO_4,_ the oxidation stages are not clearly differentiated and require further study. The anodic dissolution of EBHSS is promoted as the pH of the experiments increase, probably caused by the proton concentration in the electrolyte. The chemical dissolution of sphalerite also creates more active surfaces, which favors its electrodissolution during the anodic polarization. The macroelectrolysis experiments do not show any passivation on the mineral according to the microelectrolysis results. However, the type of electrical contact occurring in the collector causes a slow kinetic electrodissolution rate. When the temperature increases, the electrodissolution improves due to an increase in the mineral conductivity. In addition, the electrodissolution speed also increases when the EBHSS is doped, due to the created metal-semiconductor contact. Therefore, EBHSS dissolution can be carried out through electroleaching, although it is not able to generate high electrodissolution kinetics using only an external potential. To increase the electrodissolution kinetics, the process must be externally stimulated.

## Figures and Tables

**Table 1:** Mineralogical composition of zinc concentrates.

Concentrate Mineral (wt.%)	EAHSS	EBHSS
Zn	57	66
Fe	10	1
S	33	33
Total	100	100

* Data from Industrias Peñoles, personal communication.

**Table 2:** Charge densities associated to the anodic oxidation peaks of the oxidative dissolution of EBHSS.

Electrolyte	pH	Qa (mC/cm^2^) ^a^	Qa’ (mC/cm^2^) ^b^
0.1 M Na_2_SO_4_	7	122	40
0.1 M H_2_SO_4_	2	272	74
1.7 M H_2_SO_4_^c^	−0.54	2240	1660

^a^ Qa, positive direction; ^b^ Qa’, negative direction; ^c^ referred to in the text only as concentration.

**Table 3:** Comparison of the chronoampherometric and chronopotenciometric behavior of EBHSS.

Chronoampherometric Analyses		Chronopotenciometric Analyses	
E vs. SSE	J (A·cm^−2^)	E vs. SSE	J (A·cm^−2^)
1 V	1.00 × 10^−3^ (64 mA)	2 V	1.57 × 10^−3^ (100 mA)

## Data Availability

All data, belongs to this work, is given and presented herein.
